# Microbial Infections Are a Risk Factor for Neurodegenerative Diseases

**DOI:** 10.3389/fncel.2021.691136

**Published:** 2021-07-07

**Authors:** Sarah K. Lotz, Britanie M. Blackhurst, Katie L. Reagin, Kristen E. Funk

**Affiliations:** Department of Biological Sciences, University of North Carolina at Charlotte, Charlotte, NC, United States

**Keywords:** Alzheimer’s disease, amyotrophic lateral sclerosis, infection, microbes, neurodegenerative diseases, Parkinson’s disease, viruses

## Abstract

Neurodegenerative diseases, such as Alzheimer’s disease, Parkinson’s disease, and amyotrophic lateral sclerosis, comprise a family of disorders characterized by progressive loss of nervous system function. Neuroinflammation is increasingly recognized to be associated with many neurodegenerative diseases but whether it is a cause or consequence of the disease process is unclear. Of growing interest is the role of microbial infections in inciting degenerative neuroinflammatory responses and genetic factors that may regulate those responses. Microbial infections cause inflammation within the central nervous system through activation of brain-resident immune cells and infiltration of peripheral immune cells. These responses are necessary to protect the brain from lethal infections but may also induce neuropathological changes that lead to neurodegeneration. This review discusses the molecular and cellular mechanisms through which microbial infections may increase susceptibility to neurodegenerative diseases. Elucidating these mechanisms is critical for developing targeted therapeutic approaches that prevent the onset and slow the progression of neurodegenerative diseases.

## Introduction

**FIGURE 1:**
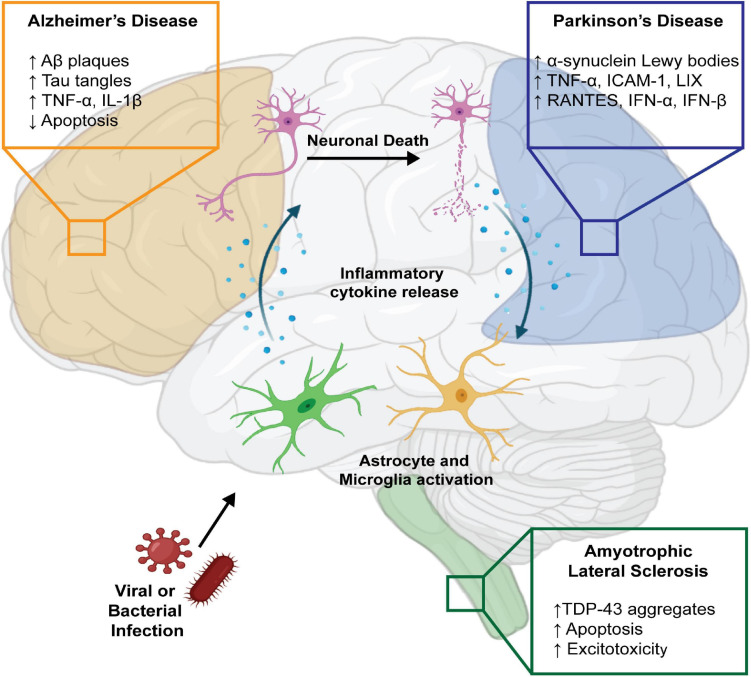
Infectious agents may contribute to neurodegenerative diseases directly or via immune activation. Infection by viral and bacterial pathogens can cause pro-inflammatory activation of CNS resident immune cells, including astrocytes and microglia, resulting in neuronal death. Additionally, cellular death directly caused by infectious agents and the release of damage-associated molecular patterns can exacerbate the inflammatory state through further activation of CNS immune cells, perpetuating a cycle of inflammation. In AD, this is often associated with high levels of pro-inflammatory cytokines TNF-α and IL-1β, reduced clearance of infected cells, and accumulation of neurotoxic aggregates composed of Aβ and Tau. This pro-inflammatory state has also been documented in the context of PD, where increased accumulation of neurotoxic α-synuclein is accompanied by high levels of TNF-α, ICAM-1, LIX, RANTES, IFN-α, and IFN-β, produced by infected and activated astrocytes and microglia. Additionally, some pathogens can directly infect neurons resulting in alterations in metabolism, enhanced neuronal excitotoxicity, and enhanced apoptosis, as seen in ALS. Created with BioRender.com.

Neurodegenerative diseases, such as Alzheimer’s disease (AD), Parkinson’s disease (PD), and amyotrophic lateral sclerosis (ALS), are clinically characterized by the progressive decline of cognitive, motor, and behavioral functions. Pathologically, these diseases exhibit significant neuronal death, brain atrophy, protein aggregation, and neuroinflammation. Despite improved understanding of disease progression, the cause or causes that initiate disease processes are not well understood. Recent genome-wide association studies have highlighted the contribution of immune molecules in many neurodegenerative diseases. Several genes with polymorphisms that increase the risk of neurodegenerative diseases, such as *CD33* and *TREM2* in AD, *PRKN, SCNA, LRRK2*, and *HLA* in PD, and *C9ORF72* in ALS have been linked to various immune functions including phagocytosis, microglial activation, complement activation, MHC class II expression, and hematopoiesis (McGeer et al., 1988; Griciuc et al., 2013; Guerreiro et al., 2013; Jonsson et al., 2013; Dzamko et al., 2015; Burberry et al., 2016; Jimenez-Ferrer and Swanberg, 2018). Because these genetic risk factors do not cause disease in all carriers, it is hypothesized that environmental factors that induce inflammation may contribute to the etiopathogenesis of neurodegenerative diseases. Microbial infections have become of increasing interest in inciting neurodegenerative pathology, as they can invade the central nervous system (CNS) and cause significant neuroinflammation through activation of resident immune cells, such as microglia and astrocytes, as well as promote infiltration of peripheral macrophages and T cells (Vasek et al., 2016; Garber et al., 2018, 2019; Klein et al., 2019). Though these immune responses exist to protect the brain, they can cause critical damage in an attempt to clear the invading pathogen (Figure 1).

Infectious agents may contribute to neurodegenerative disease pathology by eliciting an inflammatory response. Following infection, inflammation prevents damaging pathology and promotes tissue repair and regeneration; however, if uncontrolled, inflammation can become lethal to healthy cells (Chen et al., 2018). These inflammatory responses originate locally at the site of infection, but can rapidly become widespread, and in some cases, involve the CNS. Increased production of inflammatory cytokines and chemokines, including IL-1β, promote breakdown of the blood brain barrier (BBB), which typically protects the CNS resident cells from harmful agents and inflammatory mediators (Cunningham, 2013; Bendorius et al., 2018; Yong et al., 2019). However, if there is a BBB breach, these soluble mediators can stimulate CNS resident astrocytes and microglia which, upon activation, amplify inflammatory conditions in the CNS that can cause significant damage to both infected and uninfected neurons as well as resident glial cells (Holmes, 2013; Paouri and Georgopoulos, 2019; Walker et al., 2019). Importantly, neurotropic infections can lead to harmful neuroinflammation that has been identified as a potential risk factor for neurodegenerative diseases (Monastero et al., 2014; Itzhaki, 2017; Sochocka et al., 2017; Fulop et al., 2018). This review discusses recent studies linking microbial infections to neurodegenerative diseases and the cellular and molecular mechanisms through which they may increase susceptibility to disease (summarized in Table 1).

**TABLE 1:** Overview of infections associated with neurodegenerative diseases.

Disease	Pathogen	Type of pathogen	Association with disease processes
*Alzheimer’s disease (AD)*	*Chlamydia pneumoniae*	Gram-negative bacteria	• Detected in post-mortem brains and brain tissue samples of AD patients (Balin et al., 1998; Gérard et al., 2006) • Upregulated β and γ secretase in infected astrocytes, promoted Aβ peptide formation *in vitro*(Al-Atrache et al., 2019)
	*Porphyromonas gingivalis*	Gram-negative bacteria	• Detected in the brains and biofluids of AD patients (Poole et al., 2013; Dominy et al., 2019) • Increased production of TNF-α, IL-6, and IL-1β in mice (Ding et al., 2018; PMID: Ilievski et al., 2018) • Increased Aβ peptide accumulation in the brain of infected or PgLPS-treated mice (Wu et al., 2017; Ilievski et al., 2018)
	*Salmonella typhimurium*	Gram-negative bacteria	• Increased Aβ peptide deposition in the brain of infected mice to bind and entrap bacteria (Kumar et al., 2016)
	Human herpesvirus 6A (HHV-6A) and 7 (HHV-7)	Herpesvirus	• Identified HHV-6 and HHV-7 in late-onset AD patients (Readhead et al., 2018) • Reduced autophagy, promoted accumulation of hyperphosphorylated Tau and Aβ peptides (Romeo et al., 2020) • Infected microglia enhanced Aβ peptide deposition and Tau phosphorylation (Bortolotti et al., 2019)
	Herpes simplex virus 1 (HSV-1)	Herpesvirus	• Found HSV-1 antibodies in female AD patients over 60 years of age (Lövheim et al., 2015a) • Detected HSV-1 DNA in the brains of AD patients (Wozniak et al., 2009b) • Increased β-amyloidosis as an antimicrobial defense mechanism, which increased senile plaque formation (Eimer et al., 2018) • Promoted Tau hyperphosphorylation and damage in primary neurons (Zambrano et al., 2008)
	Human immunodeficiency virus (HIV-1)	Retrovirus	• Inhibited Aβ degradation in human brain cultures (Rempel and Pulliam, 2005) • Promoted cleavage of APP into Aβ peptides (Kim et al., 2013; Hategan et al., 2017) • Promoted Tau hyperphosphorylation and NFT deposition (Giunta et al., 2009). • Enhanced pro-inflammatory cytokine secretion from microglia, astrocytes, and monocytes (Nookala and Kumar, 2014; Haij et al., 2015; Liu and Kumar, 2015) • Activated immune signaling pathways (Herbein and Khan, 2008; Herbein, 2016) • Promoted Aβ secretion from primary hippocampal neurons (Aksenov et al., 2010) • Inhibited apoptosis in infected human neuroblastoma cells (Thomas et al., 2009) • Increased trafficking of Aβ to neural progenitor cells (András et al., 2017, 2020) • Detected elevated hyperphosphorylated Tau in the hippocampus of HIV-infected patients (Anthony et al., 2006)
	Human T cell leukemia virus type I (HTLV-1)	Retrovirus	• Increased activation of Tau kinases, increased Tau phosphorylation *in vitro* (Maldonado et al., 2011)

*Parkinson’s disease (PD)*	*Helicobacter pylori*	Gram-negative bacteria	• Found in PD patients in high prevalence (Huang et al., 2018; McGee et al., 2018) • Improved motor functions in patients who have cleared *H. pylori* infection (Lahner et al., 2009; Huang et al., 2018)
	Hepatitis C virus (HCV)	Flavivirus	• Increased neuronal death (Weissenborn et al., 2006; Abushouk et al., 2017; Wijarnpreecha et al., 2018) • Increased production of pro-inflammatory cytokines and chemokines from activated microglia (Lyons and Benveniste, 1998; Wu et al., 2015; Abushouk et al., 2017)
	Human immunodeficiency virus (HIV-1)	Retrovirus	• Infected dopaminergic neurons and associated with development of dementia (Nath et al., 2000)
	Cytomegalovirus (CMV)	Herpesvirus	• Elevated levels of circulating pro-inflammatory myeloid cells found in PD patients (Goldeck et al., 2016)
	Theiler’s murine encephalomyelitis virus (TMEV)	Picornavirus	• Infected dopaminergic neurons *in vivo*and promoted neurodegeneration (Oliver et al., 1997)
	Japanese Encephalitis virus (JEV)	Flavivirus	• Infected dopaminergic neurons, modulated dopamine signaling, promoted neurodegeneration (Simanjuntak et al., 2017)
	Severe acute respiratory syndrome coronavirus 2 (SARS-CoV-2)	Coronavirus	• Detected viral RNA and evidence of microglia activation and T lymphocyte infiltration in the post-mortem brain of COVID-19 patients (Matschke et al., 2020)
*Amyotrophic lateral sclerosis (ALS)*	Human immunodeficiency virus (HIV-1)	Retrovirus	• Reduced glutamate transport and increased neuronal excitotoxicity in infected human astrocytes *in vitro* (Wang et al., 2003) • Increased production and mislocalization of Fus in iPSC-derived spinal neurons (Bellmann et al., 2019) • Promoted axonal degeneration (Berth et al., 2016)
	Human endogenous retrovirus K (HERV-K)	Retrovirus	• Regulated activation of TDP-43 (Li et al., 2015)
	Theiler’s murine encephalomyelitis virus (TMEV)	Picornavirus	• Promoted TDP-43 phosphorylation, mislocalization, and aggregation following infection i*n vitro*and*in vivo*(Masaki et al., 2019)
	Rabies virus (RABV)	Rhabdovirus	• Increased production and mislocalization of Fus in iPSC-derived spinal neurons (Bellmann et al., 2019)

### Alzheimer’s Disease

AD is characterized pathologically by the deposition of two proteinaceous lesions in the brain—extracellular senile plaques and intracellular neurofibrillary tangles (NFTs) (Serrano-Pozo et al., 2011). Senile plaques are extracellular aggregates composed of insoluble amyloid beta (Aβ) peptides, the proteolytic product of amyloid beta precursor protein (APP). Under homeostatic conditions, APP is cleaved by α-secretase and γ-secretase, which is facilitated by presenilin 1 (PSEN1) (Chow et al., 2010; Murphy and LeVine, 2010; Zhang et al., 2011). In AD, APP is instead cleaved by β-secretase and γ-secretase, forming the insoluble Aβ peptides, which self-aggregate into senile plaques and are believed to be toxic to neurons (O’Brien and Wong, 2011; Kelleher and Shen, 2017; Fan et al., 2019). Neurofibrillary tangles are intracellular aggregates composed of hyperphosphorylated microtubule-associated protein Tau. Tau can be phosphorylated by a number of kinases, including cyclin-dependent kinase 5 (CDK5) and glycogen synthase kinase-3β (GSK-3β) (Gong et al., 2005). Under homeostatic conditions, phosphorylation modulates the affinity of Tau for microtubules, allowing their dynamic growth and retraction (Lindwall and Cole, 1984). In AD, Tau becomes hyperphosphorylated, which decreases its affinity for microtubules and increases its propensity to self-aggregate into pathogenic NFTs (Gong et al., 2005; Gong and Iqbal, 2008; Wang et al., 2013; Miao et al., 2019).

While the mechanisms that incite Aβ and Tau aggregation are not fully understood, recent studies have suggested a role for inflammatory cytokines, including tumor necrosis factor (TNF)-α, interferon (IFN)-γ, interleukin (IL)-1β, IL-6, IL-10, and IL-18 (Griffin et al., 1995a,b, 2006; Mrak and Griffin, 2001; Li et al., 2003). For example, IL-1β is an essential mediator of the inflammatory response and has been found to be elevated near Aβ plaques (Griffin et al., 1989; Lopez-Castejon and Brough, 2011). Expression of IFN-γ, a pro-inflammatory cytokine, was elevated in transgenic mice with AD-related pathology (Roy et al., 2020), though it does not appear to be significantly elevated in human patients (Bongioanni et al., 1997; Singh and Guthikonda, 1997). The impact of IFN-γ on AD pathology is apparently diametric, as some reports indicate that IFN-γ treatment promoted Aβ clearance by microglia and macrophages, thus reducing pathological load (Chakrabarty et al., 2010; He et al., 2020). Also, overexpression of IFN-γ in transgenic mice that develop amyloid and Tau pathologies resulted in a significant decrease in Tau pathology and improved neurogenesis, suggesting elevated levels of IFN-γ can be beneficial for alleviating AD pathology within the brain (Mastrangelo et al., 2009). However, co-stimulation of primary human astrocytes in culture with IFN-γ and TNF-α induced Aβ production, and deletion of the IFN-γ receptor reduced gliosis and amyloid plaque deposition in APP transgenic mice, which would suggest elevated levels of inflammation within the CNS exacerbates AD pathology (Blasko et al., 2000; Yamamoto et al., 2007). These seemingly conflicting observations could be, in part, due to differences in the magnitude of cytokine elevation and timeframe of expression as well as other environmental and genetic factors. Altogether, they suggest that acute episodes of neuroinflammation, such as those caused by infections, may initiate pathological Aβ and Tau deposition.

Infectious microbes have long been suspected to play a role in the onset of AD, though direct evidence is still limited (Miklossy et al., 2006; Miklossy, 2011; Bu et al., 2014). Several cohort studies have examined infectious burden in patients with AD, indicating a correlation between infections and AD pathology (Balin et al., 1998; Itzhaki and Wozniak, 2004; Gérard et al., 2006; Lövheim et al., 2015b). Using multiscale networks of AD-associated virome data, integrating genomic, transcriptomic, proteomic, and histopathological information, Readhead et al. (2018) identified evidence of increased herpesvirus 6A (HHV-6A) and human herpesvirus 7 (HHV-7) in patients with late-onset AD compared to healthy controls. Additionally, a strong association was detected between the presence of herpes simplex virus 1 (HSV-1) antibodies and patients with AD, specifically in women, subjects older than 60 years of age, and when plasma samples were taken at least 6.6 years prior to dementia diagnosis (Lövheim et al., 2015a). The authors proposed that this 6.6 year lag between HSV-1 antibody detection and AD diagnosis indicates that HSV-1 plays a role primarily in early AD development (Lövheim et al., 2015a). Furthermore, HSV-1 DNA sequences and the functional HSV-1 genome, in its entirety, were detected in the brains of patients with AD (Wozniak et al., 2009b). Similarly, the presence of *Chlamydia pneumoniae* was detected in post mortem brain-tissue samples of patients with AD (Balin et al., 1998; Gérard et al., 2006). Additionally, serum antibody levels to common periodontal microbiota were observed to increase risk of developing AD (Sparks Stein et al., 2012; Noble et al., 2014). More recent studies have identified *Porphyromonas gingivalis* in the brains and biofluids of patients with AD (Poole et al., 2013; Dominy et al., 2019). Very recently, researchers reported that in a Swedish cohort of people over the age of 50, untreated herpesvirus infection [either HSV-1 or varicella zoster virus (VZV)] increased the risk of dementia by 1.5-fold. Patients diagnosed with herpesvirus infection who took antiviral medication showed reduced risk of dementia by 25% compared to those with untreated herpesvirus infection (Lopatko Lindman et al., 2021). Epidemiological data cannot prove causation between infections and AD, but collectively these studies support the hypothesis that pathogens increase the risk of developing AD.

Some of the earliest data regarding the microbial etiology hypothesis of AD implicated HSV-1, a neurotropic enveloped virus that establishes life-long latent infection in the CNS with periodic reactivation cycles. Following resolution of primary infection, HSV-1 can remain dormant, predominantly in the trigeminal ganglion, and upon reactivation induce severe acute encephalitis in the temporal and frontal cortices of the brain, known as herpes simplex encephalitis (HSE) (Mancuso et al., 2019). Ball first proposed a link between HSV-1 and AD in 1982, recognizing that similar brain regions are affected by both HSE and AD, and that people who survived HSE exhibited clinical symptoms similar to AD, including memory loss and cognitive impairment (Ball et al., 1982). Since then substantial progress has been made to understand the molecular mechanisms by which HSV-1 may contribute to the onset of AD. Zambrano et al. (2008) showed that infection of primary neurons with HSV-1 caused significant neuronal damage and death via hyperphosphorylation of Tau, increased acetylation and tyrosination of tubulin, disrupted microtubules, and damaged and shortened neurites. Similarly, HSV-1 induced GSK3-β and protein kinase A-mediated Tau hyperphosphorylation (Wozniak et al., 2009a). All of these findings are synonymous with the pathology seen in AD, suggesting that HSV-1 infection may promote AD onset.

Another virus often associated with AD pathology is human immunodeficiency virus 1 (HIV-1), a retrovirus that can become neuroinvasive and induce severe encephalitic and cognitive changes. Patients with HIV-associated neurocognitive disorder (HAND) demonstrate increased production of Aβ and development of amyloid plaques (Antinori et al., 2007; Anthony et al., 2010; Fulop et al., 2019). HIV-1 infection induced the expression of RAGE (the receptor for advanced glycation end products) in brain endothelial cells and the accumulation of Aβ in a RAGE-dependent manner. Aβ aggregates were then transferred from brain endothelial cells to neural progenitor cells, stimulating further aggregation and progenitor cell dysfunction (Deane et al., 2003; András et al., 2010). However, while much research has focused on mechanisms of Aβ production and aggregation, the total level of Aβ in the brain also depends on the mechanisms of clearance. One clearance mechanism involves the zinc-metalloprotease neprilysin, which has been shown to cleave and degrade Aβ monomers *in vitro* and *in vivo* (Shirotani et al., 2001; Marr et al., 2004; Maruyama et al., 2005; El-Amouri et al., 2008; Grimm et al., 2013; Marr and Hafez, 2014). In an *in vitro* assay, the HIV-1 transcription transactivator (Tat) protein inhibited activity of neprilysin by 80% and increased the soluble Aβ by 125% when applied to human brain cultures (Rempel and Pulliam, 2005). HIV-1 Tat also recruited APP in lipid rafts and stimulated its cleavage by β-secretase and γ-secretase, yielding higher levels of the Aβ peptides and causing neurotoxicity (Kim et al., 2013; Hategan et al., 2017). Furthermore, HIV-1 surface protein, gp120, promoted Aβ secretion in primary embryonic rat hippocampal neurons (Aksenov et al., 2010), inhibited apoptosis of infected human neuroblastoma cells via inhibition of the Fas-pathway (Thomas et al., 2009), and induced neurotoxicity in human neuroblastoma cells through the CXCR4 and CCR5 chemokine receptors (Catani et al., 2000; Bardi et al., 2006). Additionally, HIV-1 Tat and Nef proteins exacerbated the secretion of pro-inflammatory cytokines from surrounding microglia, astrocytes and monocytes, causing neurotoxic effects (Nookala and Kumar, 2014; Haij et al., 2015; Liu and Kumar, 2015). Furthermore, Nef can mimic TNF-α signaling by activating inflammatory pathways, such as NF-κB, AP1, JNK and AKT (Herbein and Khan, 2008; Herbein, 2016). HIV-1 infection can also promote Tau aggregation, as Anthony et al. (2006) found elevated levels of hyperphosphorylated Tau in the hippocampus of HIV-1-infected individuals compared with age-matched controls. Another study found higher expression levels of the Tau kinase CDK5 in patients with HIV encephalitis versus HIV-positive patients without neuroinvasive disease, which correlated with increased Tau hyperphosphorylation (Patrick et al., 2011). Furthermore, transgenic mice that express HIV-1 glycoprotein gp120 exhibited increased brain levels of CDK5, Tau hyperphosphorylation, and neurodegeneration, which could be rescued by either genetic knockdown or pharmacological inhibition of CDK5 (Patrick et al., 2011). Additionally, HIV-1 Tat protein was similarly found to induce hyperphosphorylation of Tau in neurons through the CDK5, resulting in accelerated NFT deposition in transgenic mice (Giunta et al., 2009). Collectively, these data indicate HIV-1 infection may induce AD pathology through several potential mechanisms.

Periodontitis has been associated with increased risk of developing AD as well as other dementias (Lee et al., 2017; Kim et al., 2020). Specifically, the bacteria *P. gingivalis* and its toxic proteases, called gingipains, were identified in the brains of AD patients, and their levels correlated with AD pathology (Dominy et al., 2019). Studies investigating the mechanism underlying this relationship have identified inflammatory processes, including cytokine expression and complement activation, as well as amyloid production as mediators of *P. gingivalis* pathogenesis (Costa et al., 2021). Murine models of *P. gingivalis* infection resulted in cognitive impairment in middle-aged (12 month), but not young (4 week) mice. Researchers attributed this to elevated production of proinflammatory cytokines including TNF-α, IL-6, and IL-1β in the brains of aged mice following infection (Ding et al., 2018). This was supported by additional studies in mice and primary cell cultures of microglia and hippocampal neurons, which indicated that the lysosomal protease Cathepsin B may be critical in initiating the neuroinflammatory response to repeated *P. gingivalis* lipopolysaccharide exposure (Wu et al., 2017). Following repeated oral application of *P. gingivalis*, the bacteria was detected in the hippocampus of infected mice, serving as more direct evidence of the role of *P. gingivalis* in AD pathology (Ilievski et al., 2018). This study also showed significantly elevated expression of inflammatory cytokines IL-6, TNF-α, and IL-1β, as well as APP and β-secretase, increased Tau phosphorylation, and neurodegeneration (Ilievski et al., 2018). Together, these data propose a mechanistic link between periodontal disease and AD pathology.

Other evidence suggests that common infectious agents may contribute to AD pathology by promoting the deposition of Tau and Aβ. *C. pneumoniae* is an obligate intracellular bacterium that takes residence in the nasal and pulmonary mucosa (Porritt and Crother, 2016). It has been proposed that *C. pneumoniae* invades the brain through the lateral entorhinal cortex, then disseminates to the frontal and temporal cortices, the same regions where Aβ plaques and NFTs are found (Hammond et al., 2010; Itzhaki et al., 2016). Subsequent *in vitro* studied demonstrated that infection of astrocytes with *C. pneumoniae* decreased activity of α-secretase and increased expression of both β-secretase and γ-secretase, yielding the aggregation-prone Aβ peptide (Al-Atrache et al., 2019). Similarly, HHV-6A, a neurovirulent pathogen, was shown to promote Aβ secretion along with Tau hyperphosphorylation in primary neurons and astrocytoma cells by reducing protein degradation via autophagy and activating the unfolded protein response (Romeo et al., 2020). Furthermore, HHV-6A infection of microglial cells *in vitro* induces a pro-inflammatory activation status, stimulates the production of Aβ peptides, and promotes Tau phosphorylation (Bortolotti et al., 2019). Human T-cell leukemia virus type 1 (HTLV-1) has also been shown to increase Tau phosphorylation via CDK5 and GSK-3β activation, which resulted in neurite retraction in a cell culture model (Maldonado et al., 2011). These studies suggest that many infectious agents can contribute to AD pathology, and it is likely that the composite infectious burden is more important than a single microbe.

Antimicrobial peptides are host-defense mechanisms that defend against infectious pathogens and have recently been hypothesized to initiate pathological processes that lead to neurodegeneration. Using *C. elegans* PVD neurons as a model, researchers showed that an epidermally-expressed antimicrobial peptide NLP-29 (neuropeptide-like protein 29) causes age-dependent dendrite degeneration and that fungal infections can induce degeneration through similar mechanisms (E et al., 2018). This NLP-29-induced degeneration could be similarly stimulated in primary cultured rat neurons, suggesting that this is an evolutionarily-conserved mechanism (E et al., 2018). A recent hypothesis posits Aβ may act as an antimicrobial peptide, providing innate immune defense against infection. Soscia et al. (2010) showed that synthetic Aβ exerts antimicrobial activity *in vitro* against eight common, clinically-relevant pathogens, including seven bacterial and one yeast species. Aβ also shows neutralizing activity against seasonal (H3N2) and pandemic (H1N1) strains of influenza A virus *in vitro*, inducing viral agglutination and preventing its infectivity in epithelial cells (White et al., 2014). Bourgade et al. (2015) showed that Aβ prevented entry of HSV-1 into fibroblast, epithelial, and neuronal cell cultures. They hypothesized that based on the sequence homology between Aβ and a proximal transmembrane region of HSV-1 glycoprotein B, Aβ may directly interfere with HSV-1 replication via insertion into the viral envelope (Bourgade et al., 2015). Kumar et al. (2016) extended these findings *in vivo* to mouse and nematode models of disease, demonstrating that Aβ oligomers bind the microbial cell wall of *Salmonella typhimurium* and *Candida albicans* to prevent adhesion to host cells and reduce *S. typhimurium* load in the brains of infected mice. They went on to show a similar effect with both HSV-1 and HHV-6A infection in a mouse model of AD, demonstrating that overexpression of Aβ in mice correlated with longer survival from HSE; however, all mice still succumbed to infection within 6 days, and authors provided no evidence of reduced viral burden in the brains Aβ overexpressing mice (Eimer et al., 2018). Altogether, these data suggest that Aβ may function in innate immunity against microbial infection. However, its role in agglutination may then seed additional amyloid deposition, initiating pathogenic plaque formation, causing persistent neuroinflammation, and ultimately, lead to neurodegeneration.

### Parkinson’s Disease

PD is the second most common neurodegenerative disease, following AD, afflicting motor functions (Mhyre et al., 2012; Magrinelli et al., 2016). It is characterized by prominent dopaminergic neurodegeneration within the substantia nigra pars compacta region of the brain, which is caused by dopamine deficiency, and leads to motor neuron dysfunction (Alexander, 2004; Mhyre et al., 2012; Kalia and Lang, 2015). Patients with PD present with bradykinesia, resting tremors, gait impairment, diminished postural quality, and muscular rigidity (Antony et al., 2013; Kalia and Lang, 2015). Treatments for PD exist to manage symptoms or slow disease progression, but there is no cure. As the disease progresses, cognitive function declines and results in dementia (Aarsland et al., 2011; Kalia and Lang, 2015; Goldman et al., 2018). Though the mechanisms by which degeneration of dopaminergic neurons occurs are not fully understood, it is well established that the aggregation of misfolded α-synuclein protein in the form of Lewy bodies is a hallmark of the disease (Rocha et al., 2018). However, whether the α-synuclein aggregates themselves are neurotoxic or may be a protective mechanism to sequester the more neurotoxic protofibrils is still debated (Caughey and Lansbury, 2003). Yet another hypothesis posits that neurodegeneration is due to the loss of function of α-synuclein when it forms aggregates (Kanaan and Manfredsson, 2012). The physiological function of α-synuclein is not clear, but it appears to play an important role in dopamine biosynthesis and dopaminergic neurotransmission (Abeliovich et al., 2000; Perez et al., 2002; Liu et al., 2008). Genetic variants and post-translational modifications, including oxidation, nitration, and phosphorylation, influence the propensity of α-synuclein to aggregate; however, the physiological factors that incite these aggregation pathways are not well understood (Venda et al., 2010).

Genetic factors that cause or increase risk of developing PD include mutations in *SNCA* (encoding α-synuclein), *PRKN*, and *DJ-1*, among others (Hall et al., 2020). Interestingly, several of these genetic factors have been shown to contribute to immune defense against infectious agents. Polymorphisms in *PRKN*, a ubiquitin ligase, have been associated with increased susceptibility to intracellular pathogens, *Mycobacterium leprae* and *Salmonella typhi* (Mira et al., 2004; Ali et al., 2006). Recently PRKN was shown to limit replication of bacterial pathogens *Mycobacterium tuberculosis* and *Listeria monocytogenes* in both mice and flies by targeting them for ubiquitin-mediated autophagy (Manzanillo et al., 2013). Also, mice in which *SNCA* is deleted are more susceptible to West Nile virus and Venezuelan equine encephalitis, possibly by modulating ER stress signaling and thereby limiting viral replication (Beatman et al., 2016). In contrast, DJ-1 appears to negatively regulate the immune system. When *DJ-1* was deleted in a mouse model of polymicrobial sepsis, mice showed improved survival and bacterial clearance. Authors showed this to be due to enhanced phagocytosis and bactericidal activity in *DJ-1*-deficient macrophages, adoptive transfer of which could rescue septic wildtype mice (Amatullah et al., 2016). Although genetic mutations account for only 5–15% of all PD cases (Hall et al., 2020), better understanding these genetic causes of disease have informed the pathophysiology of the more common sporadic disease cases.

Multiple environmental factors, including chemical exposure, lifestyle, and socioeconomic conditions impact the development of PD, and pathogenic infection is increasingly recognized as a possible risk factor for PD (Bu et al., 2015; Chen and Ritz, 2018). The infectious etiology hypothesis of PD was originally proposed following the presentation of PD-like symptoms in individuals infected with influenza (Maurizi, 1987). A 1963 cohort analysis identified a striking increase in PD incidence in Guam, which seemed to recede in patients born after 1920. Authors hypothesized that this transient increase in PD incidence may have been due to the influenza pandemic of 1918 (Maurizi, 1987). Another study identified three seemingly random clusters of early-onset PD patients in Canada, in which patients lacked typical genetic markers of early-onset disease (Kumar et al., 2004). This suggested that environmental factors may have increased risk of PD in these patients, and the authors hypothesized that viral infection or other toxic exposure may be an underlying cause for these clusters of disease (Kumar et al., 2004). A cohort study examining the antibody titers to common infectious pathogens found higher seropositivity to cytomegalovirus (CMV), Epstein Barr virus (EBV), HSV-1, *Borrelia burdorferi*, *C. pneumoniae*, and *Helicobacter pylori* in PD patients compared with healthy controls (Bu et al., 2015). A recent meta-analysis of cohort and case-controlled studies revealed that patients with *H. pylori, C. pneumoniae*, Hepatitis C virus (HCV), or Malassezia yeast may be at an increased risk of PD (Wang et al., 2020). While cohort studies cannot demonstrate that infections caused PD pathogenesis, together, they suggest that infection may be an important environmental risk factor for PD.

Certain viruses directly cause degeneration of dopaminergic neurons, which results in decreased dopamine availability in the CNS. Typically considered a hepatotropic virus, HCV has recently been observed to invade the CNS and disrupt dopaminergic neurotransmission, leading to neuronal death (Weissenborn et al., 2006; Abushouk et al., 2017; Wijarnpreecha et al., 2018). HCV patients are affected by neurological complications, including cognitive impairment and peripheral neuropathy (Mathew et al., 2016). HCV may gain entry to the CNS by interacting with receptors expressed by brain microvascular endothelial cells at the BBB, including CD68, CD81, and claudin-1 (Abushouk et al., 2017; Wijarnpreecha et al., 2018). Recent studies showed that once in the CNS, HCV activated resident microglia and astrocytes. This activation promoted a pro-inflammatory state through up-regulation of cytokines and chemokines, such as TNF-α and intracellular adhesion molecule-1 (ICAM-1), which caused significant damage to dopaminergic neurons (Lyons and Benveniste, 1998; Wu et al., 2015; Abushouk et al., 2017). Additionally, HCV infection was found to indirectly trigger neurotoxic effects seen in PD through IFN-α therapy. IFN-α treatment of HCV-infected murine models inhibited transmission through the nigrostriatal dopaminergic pathway, thereby reducing the levels of dihydroxyphenylacetic acid and dopamine present in the substantia nigra (Shuto et al., 1997). Furthermore, IFN-γ, which is transcriptionally upregulated in HCV-infected human brain microvascular endothelial cells (Liu et al., 2016), caused significant death of dopaminergic neurons in both *in vitro* murine microglia/neuron co-cultures and *in vivo* murine models (Mount et al., 2007). PD is generally characterized by chronic low-level systemic inflammation; however, individuals with higher infectious burden have higher levels of circulating inflammatory cytokines, including IL-1β and IL-6 (Bu et al., 2015). PD patients infected with another Herpesviridae virus, CMV, have higher frequencies of circulating pro-inflammatory myeloid dendritic cells compared with CMV-positive subjects without PD (Goldeck et al., 2016). Furthermore, when HIV-1 becomes neuroinvasive, it shows specific affinity for dopaminergic regions, including the basal ganglia, resulting in their degeneration, decreased availability of dopamine, and the development of dementia associated with acquired immunodeficiency syndrome (AIDS) (Nath et al., 2000; Kumar et al., 2009, 2011). In a mouse model of disease, Theiler’s murine encephalomyelitis virus (TMEV) was stereotaxically inoculated into the substantia nigra. TMEV specifically infected dopaminergic neurons with minimal infection or destruction to surrounding brain regions (Oliver et al., 1997). Japanese encephalitis virus (JEV) is recognized to not only target dopaminergic neuron-rich brain regions, but can also selectively manipulate dopamine signaling to increase the cell surface expression of the molecules it uses to infect the cell (Simanjuntak et al., 2017). These studies indicate that certain viruses can specifically impact populations of neurons that can lead to neurodegeneration of dopaminergic neurons directly.

Although the CNS is a primary focus of PD research, pathophysiology affects all levels of the brain-gut axis, including the autonomic and enteric nervous systems. Mulak and Bonaz (2015) recently hypothesized that α-synuclein aggregates initiate in the gut and proceed to spread along the brain-gut axis to the CNS, resulting in the motor and neuronal deficits characteristic of PD. One pathogen hypothesized to incite α-synuclein aggregation in the gut is EBV. The C-terminal region of α-synuclein is molecularly similar to a repeat region of the latent membrane protein 1, encoded by EBV (Woulfe et al., 2014). This led to their hypothesis that in genetically susceptible individuals, antibodies to the critical repeat region of the EBV latent membrane protein may cross-react with the homologous epitope on α-synuclein and induce α-synuclein oligomerization (Woulfe et al., 2014). Following this initial aggregation event, α-synuclein oligomers may spread trans-neuronally to the CNS, causing PD neuropathology, as initially proposed by Braak and Del Tredici (2016). In support of the brain-gut trans-neuronal hypothesis, researchers showed that when pre-formed α-synuclein fibrils were injected into the duodenal and pyloric muscularis layers of a mouse model, phosphorylated α-synuclein spread to regions of the CNS affected by PD, such as the locus coeruleus and substantia nigra pars compacta (Kim et al., 2019).

Another gastric microbe that is associated with increased risk of PD is the bacteria *H. pylori* (Bu et al., 2015; Huang et al., 2018). *H. pylori* is found in the intestinal endothelium and afflicts individuals with peptic ulcers, gastritis, gastric adenocarcinoma formation, and mucosal inflammation (Camilo et al., 2017). Previous studies have linked *H. pylori* to extra-gastrointestinal diseases, such as ischemic heart disease and neurodegenerative diseases, including AD and PD (Dobbs et al., 2000, 2012; Tan et al., 2015; Huang et al., 2018; McGee et al., 2018). A Danish population-based study found that prescriptions for *H. pylori* eradication treatments and proton pump inhibitors were associated with an increased risk of PD diagnosis 5 or more years later, suggesting either that chronic *H. pylori* infection may contribute to PD etiopathogenesis or gastritis symptoms may precede pathognomonic PD symptoms (Nielsen et al., 2012). The mechanism underlying the role of *H. pylori* in PD onset is not well understood; however, the benefit of treating infections in PD patients is well-documented. A prospective cohort study found that *H. pylori*-IgG positivity in PD patients was associated with higher daily dose of levodopa and more severe symptoms compared with *H. pylori*-negative patients, and were improved after *H. pylori* eradication treatment (Mridula et al., 2017). Several studies have shown that eradicating *H. pylori* infection improved motor function of PD patients by increasing oral drug absorption (Pierantozzi et al., 2006; Lahner et al., 2009). A recent cohort study showed that PD patients with successful *H. pylori* eradication therapy exhibited improved clinical PD symptoms, including tremors, mood, and gastrointestinal distress, compared with patients with failed *H. pylori* eradication therapy (Lolekha et al., 2021). Patients with active *H. pylori* infection had longer mean levodopa onset time, suggesting that *H. pylori* may interfere with the bioavailability of levodopa, possibly because of increased gastric inflammation, delayed gastric emptying, and/or impaired active transport of levodopa to the site of absorption (Lolekha et al., 2021). Though much is still unclear of the involvement of *H. pylori* in the etiopathogenesis of PD, these data indicate that it is prevalent in PD patients and may exacerbate the symptoms of PD by interfering with levodopa bioavailability.

The global pandemic caused by severe acute respiratory syndrome coronavirus 2 (SARS-CoV-2) and its resulting disease (COVID-19) emerged as an unprecedented worldwide healthcare crisis. In the flood of viral pneumonia and the overwhelming challenges to the healthcare systems, researchers are just beginning to understand the extent to which patients develop acute or chronic neurologic manifestations. It was reported early in the pandemic that 36% of COVID-19 patients develop neurologic symptoms, but whether these were due to CNS infection, systemic inflammatory response, or intensive care unit delirium was unknown (Mao et al., 2020). More recently, a neuropathological study found evidence of viral RNA and/or protein in the brains of 53% of autopsied COVID-19 patients; however, it is important to highlight that this study analyzed only patients who died, and thus, results are probably not generalizable to less severe cases of infection (Matschke et al., 2020). In fact, a systematic search of the literature revealed that in COVID-19 patients, SARS-CoV-2 RNA was detected in only 6.4% of those who underwent cerebrospinal fluid (CSF) PCR testing, which is likely still not representative of patients with mild infection (Farooq et al., 2021; Li et al., 2021). Nonetheless, autopsies of COVID-19 patients revealed uniform presentation of neuroimmune pathology, including microglial activation and cytotoxic T lymphocyte infiltration in the brainstem and cerebellum. This pathology was independent of the detection of virus in the brain, suggesting that CNS damage and neurological symptoms may be due to cytokine storm and neuroimmune response rather than direct viral infection (Matschke et al., 2020). Considering the importance of the cerebellum and brainstem in coordinating voluntary movement, gait, posture, and motor functions, the localization of immune cell infiltration and activation may be of particular significance to the Parkinsonian symptoms seen in some post-infectious COVID-19 patients (Cohen et al., 2020; Faber et al., 2020; Méndez-Guerrero et al., 2020). Post-encephalitic parkinsonism has been reported previously for other viruses, but whether these symptoms constitute bona fide PD is unclear (Casals et al., 1998). The three case reports describing parkinsonism following COVID-19 exhibited impaired dopaminergic nigrostriatal function, but this is not necessarily diagnostic of PD (Merello et al., 2021). Rather, these may represent a transient syndrome that eventually resolves spontaneously instead of the progressive neurodegeneration of PD (Dickson, 2012). Alternatively, it is possible that SARS-CoV-2 unmasked previously preclinical PD (Merello et al., 2021). However, given the high rate of SARS-CoV-2 infection, especially in the vulnerable aging population, the potential for developing post-infectious PD is of particular concern.

### Amyotrophic Lateral Sclerosis

ALS is a neurodegenerative disease that is characterized by the loss of upper and lower motor neurons. The decrease in motor function starts as muscle weakness in the limbs and progresses to eventual paralysis of all muscular motor movements in the body (Zarei et al., 2015). Eventually, the motor neuron degeneration prevents proper functioning of the diaphragm, disrupting the proper respiratory function needed to survive (Czapliñski et al., 2003). As there are currently no pathognomonic tests for ALS, diagnosis relies on the identification of concomitant progressive upper and lower motor neuron dysfunction and the exclusion of mimicking conditions (van den Bos et al., 2019). Further complicating ALS diagnosis, is the existence of “ALS-like syndrome,” which refers to a heterogenous group of conditions in which their clinical presentation is similar to ALS (i.e., motor neuron dysfunction), but in many cases, the underlying cause of these symptoms is treatable (Ghasemi, 2016). For example, patients with HIV-1 infection presenting with ALS-like syndrome that were treated with antiretroviral therapy showed partial recovery of their motor deficit (Verma and Berger, 2006). In published reports, ALS-like syndromes cannot always be distinguished from bona fide ALS, so for the purpose of this review, we do not attempt to separate the two conditions.

There is emerging data that suggests infectious agents, including viruses and fungi, may be associated with ALS. Enteroviruses have long been suspected to play a role in ALS due to their ability to infect motor neurons in the CNS and cause meningitis and encephalitis (Ravits, 2005). However, clinical data connecting enterovirus infection and ALS have been inconclusive. Several studies have identified enterovirus RNA in spinal cord tissue of 70–80% of ALS patients (Woodall et al., 1994; Berger et al., 2000); however, others have found no detectable viral RNA in ALS patients (Swanson et al., 1995; Nix et al., 2004). Therefore, further investigation into the role of enteroviruses in ALS is necessary in order to clarify this relationship. Other recent studies have identified DNA from several *Candida* spp. of fungi, as well as fungal antigens in the CSF and brain tissue of ALS patients (Alonso et al., 2015). This, coupled with the detection of fungal hyphae within the motor cortex and spinal cord of ALS patients (Alonso et al., 2017), supports the idea that infection may contribute to or exacerbate ALS pathology. Numerous cellular dysfunctions associated with ALS are impacted by infectious agents, including protein aggregation and mislocalization, and glutamate excitotoxicity (Van Damme et al., 2017). Better understanding of how infectious agents may contribute to these cellular mechanisms that lead to motor neuron deficit will improve our understanding of the progressive neurodegeneration associated with ALS.

The presence of ubiquitinated protein aggregates in affected motor neurons is a central hallmark of disease; however, the composition of those aggregates varies among ALS patients (Blokhuis et al., 2013). The molecular characteristics and distribution of these protein aggregates, in many cases, are linked to the genetic mutations that cause the disease. However, proteinaceous aggregates of similar composition are also detected in non-mutation carriers, indicating a convergence of underlying cellular and pathological processes in both familial and sporadic cases of ALS (Blokhuis et al., 2013). The identification of ALS-associated mutations in two DNA/RNA binding proteins, TAR DNA-binding protein 43 (TDP-43) and protein fused in sarcoma (FUS), also implicate alterations in RNA processing as a key event in ALS pathogenesis (Lagier-Tourenne and Cleveland, 2009). Furthermore, mutations in TDP-43 lead to misfolded and truncated proteins, such as TDP-25 and TDP-35, as well as mislocalization from the nucleus to the cytoplasm (Prasad et al., 2019). Because the translocation of TDP-43 from the nucleus to the cytoplasm is tightly linked to the formation of pathological aggregates in the cytoplasm, it is difficult to decouple the consequences of its loss of function as a DNA-binding protein in the nucleus from the potentially toxic gain of function effects of the aggregates in the cytoplasm (Suk and Rousseaux, 2020). However, a similar cytoplasmic translocation occurs during HIV and fungal infection (Douville and Nath, 2017; French et al., 2019a). It is hypothesized that the release of neurotoxins, such as ochratoxin A, during fungal infection causes TDP-43 to mislocalize to the cytoplasm, leading to ALS pathogenesis (French et al., 2019b). The overall structure of TDP-43, along with its propensity to aggregate and mislocalize, is further influenced by post-translational modifications (François-Moutal et al., 2019). It has been demonstrated that infection with TMEV, both *in vitro* and *in vivo*, caused TDP-43 phosphorylation and cleavage, resulting in its cytoplasmic mislocalization and aggregation (Masaki et al., 2019). These data indicate that viral and fungal infections promote the neuropathology associated with ALS.

Interestingly, the relationship between viral infection and TDP-43 aggregation may be reciprocal in nature, as TDP-43 aggregation may enhance expression of endogenous retroviruses in the CNS. In autopsied samples of cortical and spinal neurons from ALS patients, the transcriptional expression of human endogenous retrovirus-K (HERV-K) polymerase was enhanced (Douville et al., 2011). Furthermore, in patients with sporadic ALS, HERV-K reverse transcriptase expression was correlated with TDP-43 and HERV-K long terminal repeats have four binding sites for TDP-43, which have been shown to regulate its activation (Li et al., 2015). In a *Drosophila* model of disease, focal glial expression of human TDP-43 triggered gypsy-ERV replication, as well as DNA damage, and neuronal apoptosis (Chang and Dubnau, 2019). Additionally, TDP-43 harbors binding sites for interferon regulatory factors (IRF) and NF-κB, which are important inflammatory mediators, causing TDP-43 to become transcriptionally upregulated in response to antiviral interferon expression (Douville et al., 2011). Together, these data suggest that HERV-K expression may be driven, in part, by TDP-43 as well as in response to local neuroinflammation (Douville et al., 2011; Li et al., 2015; Douville and Nath, 2017). In fact, TDP-43 was originally found to inhibit HIV transcription in cell culture (Ou et al., 1995), though this function is still debated and may reflect differences in cell types and model systems (Nehls et al., 2014; Manghera et al., 2016; Krug et al., 2017; Prudencio et al., 2017). Together, these studies indicate that TDP-43 aggregation and infectious agents may develop a reciprocal relationship in causing pathogenic changes that lead to ALS.

A second DNA/RNA binding protein that has been associated with familial ALS is FUS, which, when mutated, interferes with RNA metabolism, suppresses protein translation, and decreases the nonsense-mediated decay pathway (Kamelgarn et al., 2018). ALS-associated genetic mutations result in the formation of stress granules, which are composed, in part, of RNA-binding proteins, including TDP-43 and FUS (Zhang et al., 2020). The formation of FUS-containing stress granules can be stimulated by respiratory syncytial virus (RSV), as well as by poly(I:C), which is used by laboratory researchers to mimic viral double-stranded RNA (Shelkovnikova et al., 2019). It was also found that infection of induced pluripotent stem cell (iPSC)-derived spinal neurons with either rabies virus (RABV) or HIV-1 increased the production of FUS and promoted its cytoplasmic mislocalization (Bellmann et al., 2019). Furthermore, many other viruses have been shown to promote the formation of stress granules (White and Lloyd, 2012; McCormick and Khaperskyy, 2017). These studies demonstrate a link between viral infection and a key neuropathogenic hallmark of ALS.

Glutamate is a major excitatory neurotransmitter in the brain; however, excessive stimulation due to increased glutamate receptor expression or ligand availability can cause excitotoxicity and lead to neuronal death (Wang et al., 2003; Foran and Trotti, 2009). Perisynaptic astrocytes express glutamate transporters, including excitatory amino acid transporter 2 (EAAT2) and glutamate transporter-1 (GLT-1), which clear glutamate from neuronal synapses (Sheldon and Robinson, 2007). Defects in glutamate transport have been found in synaptosomes prepared from neural tissue from ALS patients (Rothstein et al., 1992). This is likely due to a combined effect of upregulation of genes that transcribe glutamate receptors in the motor cortex of ALS patients and selective loss of glutamate transporters in the motor cortex of ALS patients (Rothstein et al., 1995; Wang et al., 2006). In transgenic mice expressing mutant *SOD1*, GLT-1 was found to decrease in accordance with disease progression and survival could be extended by increasing expression of EAAT2 (Bruijn et al., 1997; Rosenblum et al., 2017). During viral infection, exposure of fetal human astrocytes *in vitro* to the HIV-1 envelope glycoprotein, gp120, caused a 40–70% decline in steady-state levels of EAAT2 RNA (Wang et al., 2003). This resulted in reduced glutamate transport and may contribute to glutamate excitotoxicity following HIV-1 infection (Wang et al., 2003). Furthermore, exposure of neurons to fungal neurotoxins caused the spontaneous release of endogenous glutamate (Bradford et al., 1990), and elevated glutamate levels have been shown to increase the toxicity associated with SOD1, as well as to promote TDP-43 translocation (Roy et al., 1998; Scofield et al., 2012). Moreover, EAAT2 expression is downregulated by TNF-α, an important cytokine involved in the antiviral response to many viruses including HIV-1, VZV, EBV, and CMV, among others (Sitcheran et al., 2005; Kim and Solomon, 2010). Thus, this excitotoxic impact from glutamate is likely common among many viral infections. Together, these data indicate that infectious diseases cause changes in glutamate signaling that can lead to excitotoxicity that is symptomatic of ALS.

## Conclusion and Future Directions

Here we have reviewed recent literature linking microbial infections to neurodegenerative diseases, including AD, PD, and ALS. Although epidemiological data indicate an association between infectious agents and neurodegenerative diseases, in many cases the molecular and cellular mechanisms underlying those associations are unclear. Alternatively, patients with neurodegenerative diseases may be at increased risk of being infected with a neurotropic agent, potentially due to compromised immune systems and/or leaky BBB in affected individuals. Further research using *in vitro* and *in vivo* models will help elucidate whether infectious agents increase the risk of developing neurodegenerative diseases on their own, via anti-microbial inflammatory pathways, or other unknown mechanisms. The study of model systems, including both rodent and non-rodent models, will also improve our understanding of post-infectious neurologic and cognitive dysfunction that occurs following many systemic and neurotropic infections beyond the common neurodegenerative diseases reviewed here. Identification of molecular mechanisms common among these neurologic disorders may lead to new diagnostic biomarkers to identify individuals that may develop progressive neurocognitive or neurodegenerative diseases, as well as new therapeutic options for them.

## Author Contributions

SL, BB, and KF wrote the manuscript. KR and SL generated the figure and table. All authors edited the manuscript.

## Conflict of Interest

The authors declare that the research was conducted in the absence of any commercial or financial relationships that could be construed as a potential conflict of interest.
